# Supplementary material for: Human subthalamic nucleus activity during non-motor decision making
Source: eLife. 2017 Dec 15;6:e31007. doi: 10.7554/eLife.31007 (PMC5780045; doi:10.7554/eLife.31007)
Supplement: Transparent reporting form [file elife-31007-transrepform.docx]

***eLife’s* transparent reporting form**

We encourage authors to provide detailed information *within their submission* to facilitate the interpretation and replication of experiments. Authors can upload supporting documentation to indicate the use of appropriate reporting guidelines for health-related research (see [EQUATOR Network](http://www.equator-network.org/%20)), life science research (see the [BioSharing Information Resource](https://biosharing.org/)), or the [ARRIVE guidelines](http://www.plosbiology.org/article/info:doi/10.1371/journal.pbio.1000412) for reporting work involving animal research. Where applicable, authors should refer to any relevant reporting standards documents in this form.

If you have any questions, please consult our Journal Policies and/or contact us: [editorial@elifesciences.org](mailto:editorial@elifesciences.org).

**Sample-size estimation**

- You should state whether an appropriate sample size was computed when the study was being designed
- You should state the statistical method of sample size computation and any required assumptions
- If no explicit power analysis was used, you should describe how you decided what sample (replicate) size (number) to use

Please outline where this information can be found within the submission (e.g., sections or figure legends), or explain why this information doesn’t apply to your submission:

Sample-size estimation computations were not conducted prior to data collection. Instead, during an 18 month period (Nov 2014-April 2016) data were collected during all DBS cases in which the patient was willing and able to participate in the task. The goal was to collect between 10-20 recording sessions during this 18 month time period. Approximately one DBS case was done per month resulting in 18 total recordings sessions. This information is stated in the methods section of the manuscript.

**Replicates**

- You should report how often each experiment was performed
- You should include a definition of biological versus technical replication
- The data obtained should be provided and sufficient information should be provided to indicate the number of independent biological and/or technical replicates
- If you encountered any outliers, you should describe how these were handled
- Criteria for exclusion/inclusion of data should be clearly stated
- High-throughput sequence data should be uploaded before submission, with a private link for reviewers provided (these are available from both GEO and ArrayExpress)

Please outline where this information can be found within the submission (e.g., sections or figure legends), or explain why this information doesn’t apply to your submission:

We captured intraoperative recordings in 18 participants undergoing deep brain stimulation (DBS) surgery of the subthalamic nucleus (STN) for Parkinson’s disease. During the operative procedure, most participants performed one session while we captured recordings from the left STN and a second session while we recorded from the right STN.  Five of the eighteen participants did not complete a second session on the opposite side because of fatigue. Thus, there were 31 total intraoperative STN recordings included in the analysis. During three of these sessions, the participant failed to correctly encode and retrieve the target numbers during several of the initial 30 blocks, but was willing to continue performing the task. In these three cases, we added additional blocks at the end of the experimental session in order to increase the total number of correct blocks included in the analysis. This information is stated in the methods section of the manuscript.

**Statistical reporting**

- Statistical analysis methods should be described and justified
- Raw data should be presented in figures whenever informative to do so (typically when N per group is less than 10)
- For each experiment, you should identify the statistical tests used, exact values of N, definitions of center, methods of multiple test correction, and dispersion and precision measures (e.g., mean, median, SD, SEM, confidence intervals; and, for the major substantive results, a measure of effect size (e.g., Pearson's r, Cohen's d)
- Report exact p-values wherever possible alongside the summary statistics and 95% confidence intervals. These should be reported for all key questions and not only when the p-value is less than 0.05.

The statistical methods used are outlined in detail in the methods section. The exact p-values for all comparisons are reported in the results section of the manuscript. The only exception to this is when very significant differences between conditions were found to have a greater value than 200 permuted differences generated by shuffling the data. In this case we simple state “p < 0.005”, which is equal to 1/200.

Please outline where this information can be found within the submission (e.g., sections or figure legends), or explain why this information doesn’t apply to your submission:

(For large datasets, or papers with a very large number of statistical tests, you may upload a single table file with tests, Ns, etc., with reference to sections in the manuscript.)

**Group allocation**

- Indicate how samples were allocated into experimental groups (in the case of clinical studies, please specify allocation to treatment method); if randomization was used, please also state if restricted randomization was applied
- Indicate if masking was used during group allocation, data collection and/or data analysis

Please outline where this information can be found within the submission (e.g., sections or figure legends), or explain why this information doesn’t apply to your submission:

This information does not apply to this submission as all subjects performed all parts of the experiment.

**Additional data files (“source data”)**

- We encourage you to upload relevant additional data files, such as numerical data that are represented as a graph in a figure, or as a summary table
- Where provided, these should be in the most useful format, and they can be uploaded as “Source data” files linked to a main figure or table
- Include model definition files including the full list of parameters used
- Include code used for data analysis (e.g., R, MatLab)
- Avoid stating that data files are “available upon request”

Please indicate the figures or tables for which source data files have been provided:

The raw electrophysiology data and relevant code will be made available here:

<https://neuroscience.nih.gov/ninds/zaghloul/downloads.html>
